# *C9orf72* arginine-rich dipeptide proteins interact with ribosomal proteins in vivo to induce a toxic translational arrest that is rescued by eIF1A

**DOI:** 10.1007/s00401-018-1946-4

**Published:** 2019-01-02

**Authors:** Thomas G. Moens, Teresa Niccoli, Katherine M. Wilson, Magda L. Atilano, Nicol Birsa, Lauren M. Gittings, Benedikt V. Holbling, Miranda C. Dyson, Annora Thoeng, Jacob Neeves, Idoia Glaria, Lu Yu, Julia Bussmann, Erik Storkebaum, Mercedes Pardo, Jyoti S. Choudhary, Pietro Fratta, Linda Partridge, Adrian M. Isaacs

**Affiliations:** 1 Department of Neurodegenerative Disease, UCL Institute of Neurology, Queen Square, London, WC1N 3BG UK; 2 Department of Genetics, Evolution and Environment, Institute of Healthy Ageing, UCL, Darwin Building, Gower Street, London, WC1E 6BT UK; 3 UK Dementia Research Institute at UCL, Cruciform Building, London, WC1E 6BT UK; 4 Department of Neurosciences, Experimental Neurology, KU Leuven – University of Leuven, Leuven Brain Institute (LBI), Leuven, Belgium; 5 VIB, Center for Brain & Disease Research, Laboratory of Neurobiology, Leuven, Belgium; 6 Sobell Department of Motor Neuroscience and Movement Disorders, UCL Institute of Neurology, Queen Square, London, WC1N 3BG UK; 7 Wellcome Trust Sanger Institute, Wellcome Trust Genome Campus, Hinxton, Cambridge, UK; 8 Present Address: Division of Cancer Biology, The Institute of Cancer Research, 123 Old Brompton Road, London, SW7 3RP UK; 9 Molecular Neurogenetics Laboratory, Max Planck Institute for Molecular Biomedicine, 48149 Münster, Germany; 10 Faculty of Medicine, University of Münster, 48149 Münster, Germany; 11 Department of Molecular Neurobiology, Donders Institute for Brain, Cognition and Behaviour, Radboud University, Nijmegen, The Netherlands; 12 MRC Centre for Neuromuscular Disease, UCL Institute of Neurology, Queen Square, London, WC1N 3BG UK; 13 Max Planck Institute for Biology of Ageing, Joseph-Stelzmann-Strasse 9b, 50931 Cologne, Germany

**Keywords:** ALS, FTD, Drosophila, Dipeptide, C9orf72

## Abstract

A GGGGCC hexanucleotide repeat expansion within the *C9orf72* gene is the most common genetic cause of both amyotrophic lateral sclerosis and frontotemporal dementia. Sense and antisense repeat-containing transcripts undergo repeat-associated non-AUG-initiated translation to produce five dipeptide proteins (DPRs). The polyGR and polyPR DPRs are extremely toxic when expressed in *Drosophila* neurons. To determine the mechanism that mediates this toxicity, we purified DPRs from the *Drosophila* brain and used mass spectrometry to identify the in vivo neuronal DPR interactome. PolyGR and polyPR interact with ribosomal proteins, and inhibit translation in both human iPSC-derived motor neurons, and adult *Drosophila* neurons. We next performed a screen of 81 translation-associated proteins in GGGGCC repeat-expressing *Drosophila* to determine whether this translational repression can be overcome and if this impacts neurodegeneration. Expression of the translation initiation factor eIF1A uniquely rescued DPR-induced toxicity in vivo, indicating that restoring translation is a potential therapeutic strategy. These data directly implicate translational repression in *C9orf72* repeat-induced neurodegeneration and identify eIF1A as a novel modifier of *C9orf72* repeat toxicity.

**Electronic supplementary material:**

The online version of this article (10.1007/s00401-018-1946-4) contains supplementary material, which is available to authorized users.

## Introduction

A GGGGCC hexanucleotide repeat expansion within the *C9orf72* gene is the most common genetic cause of both amyotrophic lateral sclerosis (ALS) and frontotemporal dementia (FTD). Toxicity has been proposed to arise in patients either due to haploinsufficiency of the *C9orf72* gene, or to two major gains of function mechanisms. These are the production of sense and antisense repeat RNA, leading to the formation of RNA-binding protein sequestering RNA foci, or the translation of the repeat RNA into highly repetitive dipeptide proteins [30]. Translation of repeat RNA occurs in the sense and antisense direction in an AUG-independent manner, known as repeat-associated non-AUG-initiated translation (RAN translation) [50], leading to the production of five dipeptide proteins (DPRs): polyGP, polyGA and polyGR from the sense strand [1, 31], and polyGP, polyPA and polyPR from the antisense strand [12, 51].

Whether or not repeat RNA leads to toxicity in humans is currently contentious [30]. However, several groups, including our own, have previously demonstrated that expression of ATG-driven arginine-containing dipeptide proteins (polyGR, polyPR), which have been recodonised to lack a repetitive RNA intermediate, is extremely toxic to various model systems including human cell lines, *Drosophila* and mice [18, 28, 43, 48], whilst polyGA exerts toxicity in some model systems [21, 26, 36, 49].

The mechanisms by which the arginine-containing dipeptides are toxic is currently unresolved, with several theories posited including the induction of nucleolar dysfunction [18], binding to cellular RNAs [16], or disruption of nucleocytoplasmic transport [6, 10, 15, 46, 47] having been suggested as non-mutually exclusive mechanisms. Recently, several groups have published interactomes of DPR proteins derived from human cell lines and have commonly observed ribosomal proteins and RNA-binding proteins as key constituents [5, 14, 16, 20, 22, 23, 45].

Here, we have identified proteins interacting with arginine-containing DPRs in fly brains, by purifying dipeptide proteins directly from *Drosophila* neurons using tandem affinity purification coupled to mass spectrometry. Consistent with previous results, we identified large numbers of ribosomal proteins binding to polyPR and polyGR, suggesting that translation may be impaired by the arginine-rich DPRs. Consistently, we observed reduced translational activity in human induced pluripotent stem cell (iPSC)-derived motor neurons (MNs) overexpressing arginine-rich DPR proteins, as well as in our in vivo *Drosophila* models expressing recodonised arginine-rich DPRs, or GGGGCC repeat RNA. To identify factors involved in protein translation that could rescue toxicity, we performed an extensive genetic screen of ribosomal proteins and translation initiation factors in our GGGGCC repeat *Drosophila* model. We identified a single translation-associated gene, the translation initiation factor eIF1A, as capable of rescuing neuronal toxicity by enhancing translation. These findings demonstrate that translational repression is a crucial toxic mechanism in an in vivo model system and identify a novel regulator of this process.

## Materials and methods

### *Drosophila* stocks and maintenance

*Drosophila* stocks were maintained on SYA food (15 g/L agar, 50 g/L sugar, 100 g/L autolysed yeast, 30 ml/L nipagin (10% in ethanol) and 3 ml/L propionic acid) at 25 °C in a 12-h light/dark cycle with constant humidity. The elavGS stock was generously provided by Herve Tricoire (Paris Diderot University) [33]. The GMR-Gal4 line was obtained from the Bloomington *Drosophila* Stock Centre. UAS-GR50-FLAG, UAS-PR50-FLAG and UAS-GA50-FLAG flies were kindly provided by Ludo Van Den Bosch (Vlaams Instituut voor Biotechnologie) [6]. The UAS-metRS^L262G^-EGFP line was described previously [9]. UAS-36R, UAS-GR100, and UAS-PR100 flies have been previously described [28]. The indicated overexpression lines were obtained from the FlyORF collection, including the UAS-eIF1A line (F001317) [4].

### Generation of transgenic *Drosophila*

GA, GR and PR coding sequences were amplified without their original 3′ stop codon from the original pUAST attb plasmids containing 200-aa dipeptide sequences (described in [28], using a common forward primer and a sequence-specific reverse primer. PCR products were digested and ligated into the GS-CTAP plasmid [19]. Vectors were sequenced prior to injection into *Drosophila*. Repeat lengths were 199 amino acids for GR and GA, and 179 amino acids for PR. Plasmids were injected by the University of Cambridge, Department of Genetics Fly Facility.

### Assessment of eye phenotypes

Flies carrying UAS-dipeptide-GSTAP constructs were crossed to the GMR-GAL4 driver line. The resulting cross was allowed to develop and eclose at 25 °C; female eyes were photographed on the day of emergence.

### *Drosophila* lifespan assays

The parental generation of the genotype indicated in each lifespan assay was allowed to lay for 24 h on grape agar plates supplemented with yeast. Eggs were deposited at a standard density into bottles containing SYA medium. Adult experimental flies were allowed to emerge and mate for 2 days before being lightly anaesthetised with CO_2_, and females randomly allocated onto SYA containing RU486 (200 μM) at a standard density per vial, with a minimum 100 flies per condition. At regular intervals (see results), flies were tipped onto fresh food and dead flies counted. Escaping flies were censored from the data. Lifespans are presented as cumulative survival curves. Significance was assessed using the log rank test.

### Tandem affinity purification of dipeptides

Experimental flies were bred as described for lifespans to generate the following genotypes: w; UAS-GR-GSTAP/+; elavGS/+w; UAS-GA-GSTAP/+; elavGS/+w; +; UAS-PR-GSTAP/elavGS.

Newly emerged flies were allowed to mate for 3–4 days before being tipped onto SYA medium containing 200 μM RU486 in bottles. Flies were left on RU486 for 3 days before being snap frozen in liquid nitrogen and stored at − 80 °C. Tandem affinity purification was performed as previously described, with modifications [40]. Approximately 1 g of heads was ground in a mortar on dry ice before being transferred to a chilled 15 ml Dounce homogeniser and being homogenised using a loose pestle in ice cold 8 ml lysis buffer (50 mM Tris–HCl (pH 7.4), 125 mM NaCl, 5% glycerol, 1.5 mM MgCl, 25 mM NaF, 0.2% IGEPAL, 1 mM NaVO_4_, 1 mM DTT, 1 mM EDTA). Homogenates were centrifuged at 21,000×*g* for 30 min at 4 °C, and supernatant was centrifuged again under the same conditions. For each sample, 400 μl of IgG Sepharose 6 Fast Flow beads (GE Healthcare) was washed three times in an excess IgG wash buffer (10 mM Tris–HCl (pH 8.0), 150 mM NaCl, 0.1% IGEPAL). Cleared homogenates were incubated with IgG Sepharose beads for 2 h at 4 °C with agitation. The homogenate and bead mixture was loaded onto a 15-ml econocolumn (Biorad) at 4 °C. The homogenate was drained, and beads were washed three times with 10 ml of ice cold IgG wash buffer, before being washed once with 10 ml TEV cleavage buffer (10 mM Tris–Cl pH 8.0, 150 mM NaCl, 0.1% IGEPAL, 0.5 mM EDTA, 1 mM DTT). Following this, TEV cleavage buffer containing 100 units/ml of AcTEV enzyme (Thermo Fisher Scientific) was applied, and beads were incubated in the column with agitation at 18 °C for 2 h.

240 μl of Streptavidin Agarose beads (Pierce) were washed three times in TEV cleavage buffer. The TEV cleavage product was incubated with the Streptavidin beads for 1.5 h at 4 °C. Beads were then washed once with TEV cleavage buffer, and then three times in TEV cleavage buffer without IGEPAL.

Beads were resuspended in 10 mM Tris–HCl pH 8.0, 150 mM NaCl and split in two; each sample was resuspended in 10 mM Tris–HCl pH 8.0 and 150 mM NaCl with 10 mM TCEP, and incubated at 37 °C for 10 min with shaking. Beads were then pelleted and TCEP solution removed and replaced with 10 mM Tris–HCl pH 8.0, 150 mM NaCl with 20 mM iodoacetamide and incubated in the dark at room temperature for 30 min with shaking. Beads were pelleted and resuspended in 100 μl of 50 mM ammonium bicarbonate with 0.2 μg of sequencing-grade trypsin (Roche) (reconstituted in 0.5% formic acid) and digested overnight. After digestion, the beads were pelleted and the liberated peptides removed from the beads.

The peptide-containing supernatant was placed in a fresh Eppendorf tube, and the trypsin reaction was halted by the addition of 5% formic acid to a final concentration of 0.25%. To liberate residual peptide, beads were resuspended in 100 µl of 1 M ammonium bicarbonate solution before being pelleted as before. Supernatant was removed, and 5% formic acid was added to a final concentration of 0.25%. For each sample, the original supernatant was pooled with the wash. Samples were frozen overnight at − 20 °C before being dried in a speed vacuum at 45 °C.

### Mass spectrometry

All samples were filtered through in-house-made C8 tips (Empore Octyl C8, 3 M) to remove residual particulate material. Peptides were resuspended in 0.5% formic acid/100% H_2_O before LC–MS/MS analysis on an Ultimate 3000 RSLCnano System coupled to a LTQ Orbitrap Velos hybrid mass spectrometer equipped with a nanospray source. The sample was first desalted on a PepMap C18 nano trap (100 μm i.d. × 20 mm, 100Å, 5 μm) at 10 μL/min for 15 min, then separated on a PepMap RSLC C18 column (75 μm i.d. × 250 mm, 100 Å, 2 μm) with a flow rate of 300 nl/min in a linear gradient of 4–32% CH_3_CN/0.1% formic acid in 90 min, with total 130 min of acquisition time. The HPLC, columns and mass spectrometer were all from Thermo Fisher Scientific. The Orbitrap mass spectrometer was operated in the standard “top 15″ data-dependent acquisition mode while the preview mode was disabled. The MS full scan was set at *m*/*z* 380–1600 with the resolution at 30,000 at *m*/*z* 400 and AGC at 1 × 10^6^ with a maximum injection time at 200 ms. The siloxane ion at 445.120020 was used as lock mass. The 15 most abundant multiply charged precursor ions (*z* ≥ 2), with a minimal signal above 3000 counts, were dynamically selected for CID (collision induced dissociation) fragmentation in the ion trap, which had the AGC set at 5000 with the maximum injection timemat 100 ms. The precursor isolation width was set at 2 Da. The normalised collision energy for CID MS/MS was set at 35%. The dynamic exclusion duration time for selected ions for MS/MS was set for 60 s with ± 10 ppm exclusion mass width.

The raw files were processed with Proteome Discoverer v1.4 (Thermo). Database searches were performed with Mascot (Matrix Science) against the *Drosophila* Uniprot database (v. December 2015). The search parameters were: trypsin digestion, two missed cleavages, 10 ppm mass tolerance for MS, 0.5 Da mass tolerance for MS/MS, with variable modifications of carbamidomethyl (C), N-acetylation (protein), oxidation (M), and pyro-glu (NtermQ). Database search results were refined through Mascot Percolator (FDR < 1%). High-confidence peptides were apportioned to proteins using Mascot Protein Family summary. Protein identification required at least one high-confidence peptide (FDR < 1%) and a minimum Mascot protein score of 20. External contaminants were removed for further analysis. The mass spectrometry proteomics data have been deposited to the ProteomeXchange Consortium via the PRIDE [41] partner repository with the dataset identifier PXD012099​.

### FUNCAT

To ensure equivalent baseline translation, repeat insertions were backcrossed for six generations into the w1118 background. The elavGS driver and the UAS-metRS^L262G^-EGFP insertion were recombined onto the same chromosome, and UAS-repeat stocks or w1118 crossed to this stock. Adult females were split into vials containing SYA media supplemented with 200 μM RU486. 3 days after induction, flies were briefly anaesthetized using CO_2_ and injected into the haemolymph with 32.2 nl of 200 mM of azidonorleucine (ANL) solution made in sterile PBS (pH7.4) or vehicle alone. Flies were allowed to recover for 48 h on food supplemented with RU486 before adult brains were dissected in haemolymph-like solution with calcium, and fixed and processed in the manner described previously [9]. Following the FUNCAT procedure, brains were mounted in vectashield with DAPI (Vectorlabs). Images of neurons were taken using a Zeiss LSM 710 confocal microscope with the 63× objective lens. An image was taken in the optic lobe of each brain, three boxes of equal size were drawn in different regions of the tissue using the GFP channel as a guide, and TAMRA intensity measured using imageJ image analysis software, GFP intensity and TAMRA intensity were averaged across the three regions. Experimenter was blinded to genotype during image acquisition and analysis.

### Generation of GFP-tagged DPR and FLAG-EIF1AX plasmids

Previously described 200-aa-encoding dipeptide repeat sequences (GA100, PR100, and GR100) [28] were subcloned into the PCDNA3.1(+) vector using BamHI and NotI restriction sites. Following this, the GFP coding sequence was liberated from the pEGFP-C3 vector (Addgene 6082-1) using the BglII and BmtI restriction sites, and was subcloned N-terminally to the dipeptide coding sequence by digesting the PCDNA3.1(+)-DPR vector with BamHI and BmtI. Human *EIF1AX* coding sequence was generated by gene synthesis (GeneArt) inserted into the PCDNA3.1(+) plasmid and an N-terminal FLAG tag added by PCR.

### Cell culture and AHA staining

For iPSC culture, an iPSC line from a healthy donor was maintained in Essential E8^™^ medium cultured on Getrex-coated plates. The control iPSC line has been used in previous study described as ‘Control 1′, the healthy donor was male and 64 years old at time of biopsy [38]. iPSCs were grown to full confluence and then induced into motor neuron-like cells as previously described [13, 38]. Briefly, after the patterning stage, at day 18 of differentiation, progenitor MNs were expanded in neuronal medium containing FGF (10 ng/µl).

Progenitor MNs were cultured with additional 10 µM, Y-27632 dihydrochloride (ROCK inhibitor), for 1 h prior to nucleofection. Cells were lifted into suspension after incubation for 5 min at 37 °C with 0.5 mM EDTA in 1× PBS. 2 × 10^6^ cells per condition were centrifuged at 300×*g* for 5 min. Cell pellet was gently resuspended in 100 µl of Basic Neuron Nucleofector^™^ solution (Lonza). 4 µg of plasmid DNA was added before the mixture was transferred into cuvette and electroporated using Amaxa™ Nucleofector™ II device and programme A-033. Immediately afterwards, the mix was added to pre-warmed neuronal medium containing 0.1 µM Compound E (Merk, 565790) and 10 µM ROCK inhibitor. Cells were then plated onto coverslips pre-coated with Geltrex™.

24 h after nucleofection cells were placed in neuronal methionine-free medium for 30 min, made by supplementing DMEM–l-methionine–l-cysteine (Gibco^™^ 21013024) with 0.23 mM sodium pyruvate, 10 mM HEPES, 0.26 mM l-cysteine, 0.067 mM l-proline, 0.674 µM zinc sulphate, 5 nM B12, non-essential amino acids 0.5% (Gibco), Glutamax 1%, 5 µg/ml insulin, B27 supplement (1×) and N2 supplement (1×). Cells to be used as negative control had neuronal methionine-free medium supplemented with 50 µM anisomycin. Cells were then cultured with 150 µM Click-iT^®^ AHA (L-azidohomoalanine) (Thermo Fisher), in neuronal methionine-free medium for 2 h. Negative controls had 150 µM Click-iT^®^ AHA and additional 100 µM anisomycin. Cells were fixed and stained with Click-iT^®^ Cell Reaction Buffer Kit (Cat. no. C10269) and the fluorophore Alexa 555 alkyne (1 µM).

HeLa cells were maintained in DMEM complete medium (10% FBS, 1% Glutamax, 1% sodium pyruvate, 1% Pen/Strep) and plated on coverslips in 24-well plates the day before transfection. Cells were transfected with either GFP alone (320 ng), GFP-GR100 (80 ng) together with FLAG-EIF1AX (240 ng), or GFP-GR100 (80 ng) together with FLAG alone (240 ng), using Lipofectamine 2000. After 24 h, the medium was changed to methionine-free DMEM for 30 min before the addition of AHA as described above for MN progenitors. Cells were stained with anti-FLAG antibody (F1804, Sigma) prior to imaging.

A confocal microscope (Zeiss LSM 710 for MN progenitors, Zeiss LSM 880 for HeLa cells) was used to take Z stacks and maximum intensity projection images were analysed using ImageJ to quantify the intensity of AHA staining (Alexa 555) in cells positive for GFP or GFP-tagged DPRs.

### *Drosophila* polyGR ELISA assay

Heads from female flies induced on SYA medium containing 200 μM RU486 for 7 days were collected and processed in the manner described previously [29].

### Quantitative reverse transcription PCR (RT-qPCR)

Female flies were induced on SYA medium containing 200 μM RU486 for 5 days before being flash frozen in liquid nitrogen. 9–10 heads per replicate were extracted using TRIzol reagent (Thermo Fisher Scientific) following manufacturer’s protocol. 1 μg of RNA per sample was treated with DNase I (Ambion), followed by reverse transcription using the SuperScript II system (Invitrogen) using random hexamers (Thermo Fisher Scientific). Quantitative PCR was performed using the QuantStudio 6 Flex Real-Time PCR System (Applied Biosystems) using SYBR^®^ Green master mix (Applied Biosystems). Values were obtained using the relative standard curve method and normalised to alphaTub84B. Primers used were: EGFP forward: GGTGAACTTCAAGATCCGCC; EGFP reverse: CTTGTACAGCTCGTCCATGC; Tub84B forward: TGGGCCCGTCTGGACCACAA; Tub84B reverse: TCGCCGTCACCGGAGTCCAT.

### Bioinformatics

The string network was created by uploading GR and PR-interacting proteins to the STRING web interface [39], and the resultant image was created using Cytoscape. Gene ontology analysis was performed using WebGestalt [42].

To assess overlap of mass spectrometric data sets, data sets were downloaded from supplementary material of the referenced original papers: from Lee et al. (2016) [20] GR and PR interactors with a Saint cutoff of > 0.9 were taken; from Lin et al. [22] the PR interactome common to both methods was used; for Boeynaems et al. [5] the PFA crosslinked data set was used; for Lopez-Gonzalez et al. [23] the non-RNAse-treated data set was used; the Yin et al. [45] dataset was used, with removal of the ten proteins not included in the analysis performed in the original study. Genes were converted to *Drosophila* orthologs using BioMart (Ensembl), and significance of overlap performed in comparison with the hypergeometric distribution in R based on an estimated *Drosophila* protein-coding genome size of 13,931 genes. The six-way overlap between the data sets was created using jVenn [2].

### *Drosophila* screen

Flies carrying UAS-ribosomal and initiation factor protein transgenes inserted at the attP-86Fb integration site were ordered from the FlyORF stock repository and crossed to virgin females carrying the UAS-36R and elavGS transgenes. The attP-86Fb injection strain was crossed as the control. Emerging adult flies were allowed to mate for 24 h, before 100 flies of each genotype were split at a density of 25 flies per vial on SYA supplemented with 200 μM RU486. Flies were scored until days 12–13 (approximately 30–40% control flies had died) and mortality of each line scored as a percentage of controls. Transgenes leading to less than 30% death, relative to the control, were rescreened using a similar protocol. If the same effect was observed, transgenes were backcrossed for six generations in to the w1118 background and retested.

## Results

### Characterisation of arginine-rich DPR interactome in vivo in *Drosophila* neurons

To determine which proteins the arginine-containing DPRs form complexes with in an intact neuronal system, DNA sequences encoding recodonised ATG-driven dipeptide proteins [28] were cloned upstream of a tandem affinity purification (TAP) tag containing a protein G module and streptavidin-binding domain separated by an internal tobacco etch virus (TEV) protease cleavage site (GSTAP tag). We generated *Drosophila* capable of expressing 90–100 repeats of the following DPRs: polyGR, polyPR and polyGA. After generation of transgenic *Drosophila*, constructs were expressed in the developing compound eye using the GMR-Gal4 driver and, as expected, strong toxicity was observed for the GR-GSTAP and PR-GSTAP constructs but not the GA-GSTAP control construct (Suppl. Figure 1a, Online Resource 1). We confirmed expression of the GSTAP-tagged constructs using the elavGS driver which drives expression in adult neurons, observing a robust expression of polyGA and a weaker expression of polyGR and polyPR (Suppl. Figure 1b, Online Resource 1).

**Fig. 1:**
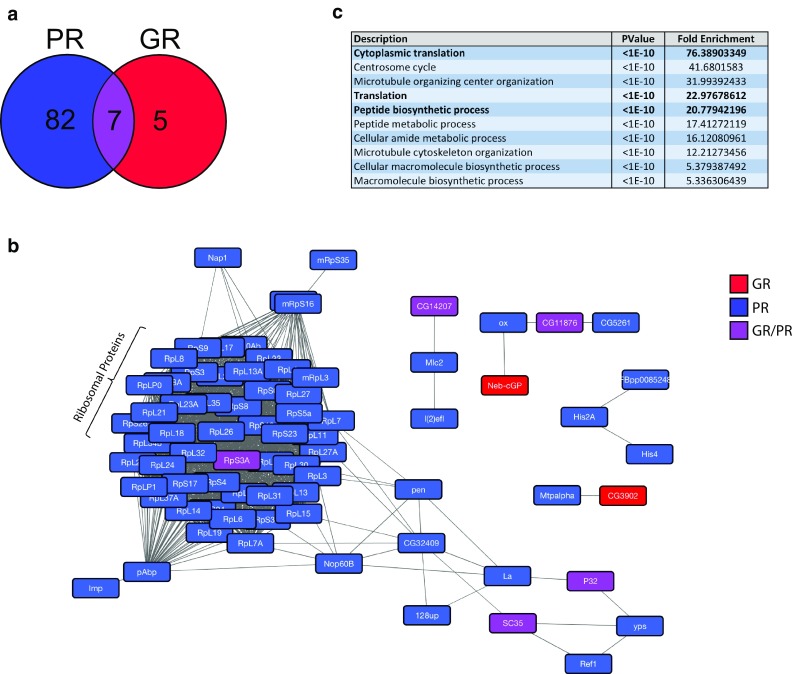
Arginine-rich DPRs bind to ribosomal proteins and proteins involved in translation. **a** Results of mass spectrometric identification of polyGR- and polyPR-interacting proteins in vivo. Numbers represent individual proteins identified in a minimum of 2/3 replicates. A consistently larger number of interactors were identified as binding specifically to PR (82/94), compared to GR, where (5/94) interactors were specific to GR, and (7/94) interactors were specific to both data sets. **b** STRING analysis was used to search for high-confidence-known protein complexes amongst arginine-rich DPR interactors. Proteins depicted in red were specific to polyGR, proteins depicted in blue were specific to polyPR, and proteins depicted in purple were common to both data sets. Large numbers of ribosomal proteins were identified as binding to polyPR, as well as Rps3a which was found to bind to both polyGR and polyPR. **c** Top ten significantly overrepresented biological process gene ontology terms associated with proteins identified as binding polyPR and/or polyGR. Notably, cytoplasmic translation, translation and peptide biosynthetic process were significantly enriched categories (highlighted in bold) (*P* < 1E−10 for all)

We performed tandem affinity purification coupled to LC–MS/MS to identify dipeptide-interacting proteins in adult *Drosophila* neurons, performing three independent replicates and including only data present in ≥ 2/3 replicates. We used polyGA as a negative control, filtering the results to exclude polyGA-interacting proteins, as these probably represent proteins that bind to the TAP tag itself, or proteins that are unlikely to play a role in arginine-rich DPR toxicity in *Drosophila*. In total, we identified 94 proteins that bind to either polyPR or polyGR, with 82 specific to polyPR alone, 5 specific to polyGR alone, and 7 common between both proteins (Fig. 1a). The higher number of interactors in the polyPR data set was consistent across replicates (Suppl. Table 1, Online Resource 2), suggesting that polyPR may bind to protein complexes with a higher degree of avidity, or may be expressed at a higher level/undergo less clearance compared to polyGR. 49 proteins were found to overlap with both polyGA and polyPR and/or polyGR (Suppl. Figure 2, Online Resource 1), which are likely to have interacted with the epitope tag. Two proteins were found to be specific to GA, including unc-119, which has previously been demonstrated to be a key GA-interacting protein in transfected cells and forms aggregates in *C9orf72* human post-mortem tissue [26, 37].

Several recent studies have performed mass spectrometry on PR- or GR-interacting proteins using lysates from immortalised non-neuronal cell lines [5, 16, 20, 22, 23, 45]. To determine whether these shared common interactors with our dataset, where a complete list of interactors was available, we converted the identified human genes into *Drosophila* orthologs, and analysed the degree of overlap, finding a highly significant overlap with each data set (Suppl. Figure 3, Online Resource 1). We performed STRING analysis to cluster the *Drosophila*-interacting proteins into a network, and observed a strong enrichment of ribosomal proteins in the *Drosophila* data set (Fig. 1b). When gene ontology analysis was performed on the total set of interacting proteins, the most significantly overrepresented ontology term was “cytoplasmic translation” (*P* < 1E−10). Notably, this was consistent with the human data set, where of the orthologous shared interactors present in all six data sets (five human cell line and one *Drosophila* neuron), 100% were ribosomal proteins, with both small and large ribosomal subunits represented (Suppl. Figure 4, Online Resource 1).

### Expression of arginine-rich dipeptide proteins suppresses translation in human iPSC-derived motor neurons

**Fig. 2:**
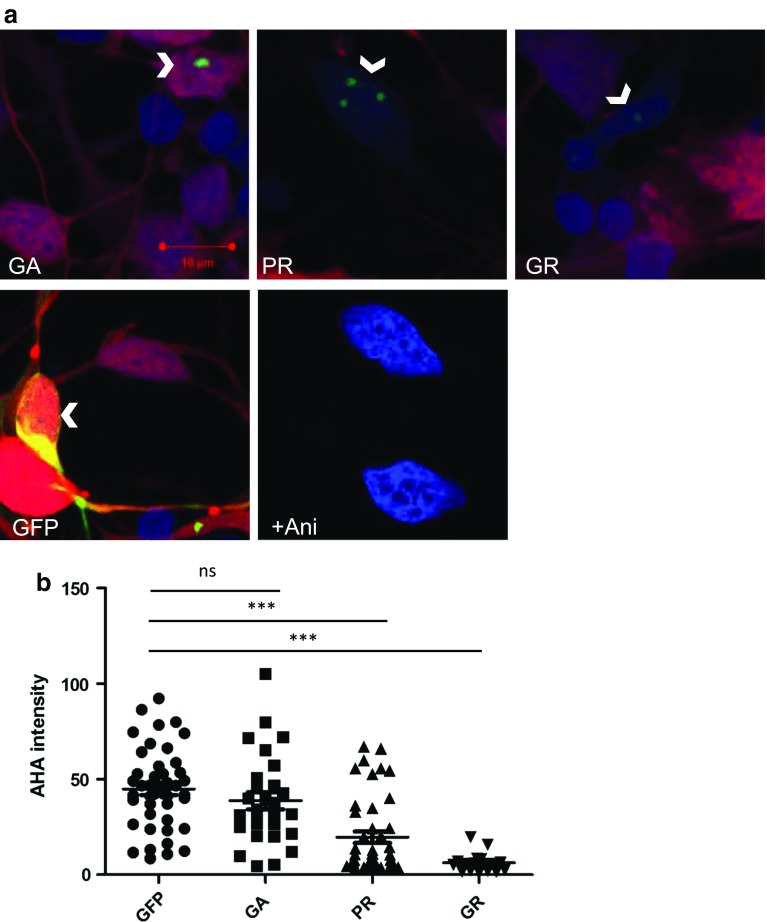
Expression of arginine-rich DPRs suppresses translation in human iPSC-derived MNs. **a** iPSC-MNs transfected to express DPRs (GFP-GA100, GFP-PR100, GFP-GR100) or GFP alone (arrow heads, green) were treated with AHA and newly synthesised proteins labelled with alkyne-tagged Alexa 555 (red), cells were counterstained with DAPI (blue). iPSC-MNs treated with 100 µM of anisomycin and AHA for 2 h were used as a negative control for AHA imaging (+Ani) and did not show incorporation of AHA (red). **b** AHA intensity was measured in DPR- or GFP-positive cells. Two inductions of control iPSC-MNs were nucleofected, from which *n* = 44, 28, 43, 15 for GFP-, GA-, PR- or GR-positive iPSC-MNs, respectively, were analysed. Error bars are mean ± SEM. Kruskal–Wallis test and Dunn’s Multiple comparison test, ***P < 0.001, ns indicates not significant

The interaction of the arginine-rich DPRs with ribosomal subunits suggested that they might be capable of interfering with the process of translation. We, therefore, transfected iPSC-derived motor neuron progenitors with plasmids encoding the N-terminally tagged arginine-rich DPR proteins GFP-GR100 and GFP-PR100 with GFP-GA100 or GFP alone as controls. We assessed translation by starving cells of methionine, and feeding them with a pulse of L-azidohomoalanine (AHA) 24 h after transfection with DPRs. Following fixation, a fluorescent label was covalently linked to the AHA-containing proteins, allowing assessment of the level of translation that had occurred. As expected, we observed robust incorporation and labelling of AHA in GFP-transfected cells, and this incorporation is prevented by the addition of the protein synthesis inhibitor anisomycin (Fig. 2a). Furthermore, when we assessed the level of translation in cells expressing DPR constructs we found that, whilst GFP-GA100 had no effect on translational activity, both GFP-GR100 and GFP-PR100 strongly and significantly reduced translation in human neurons (Fig. 2a, b).

### Expression of arginine-rich dipeptide proteins suppresses translation in vivo

**Fig. 3:**
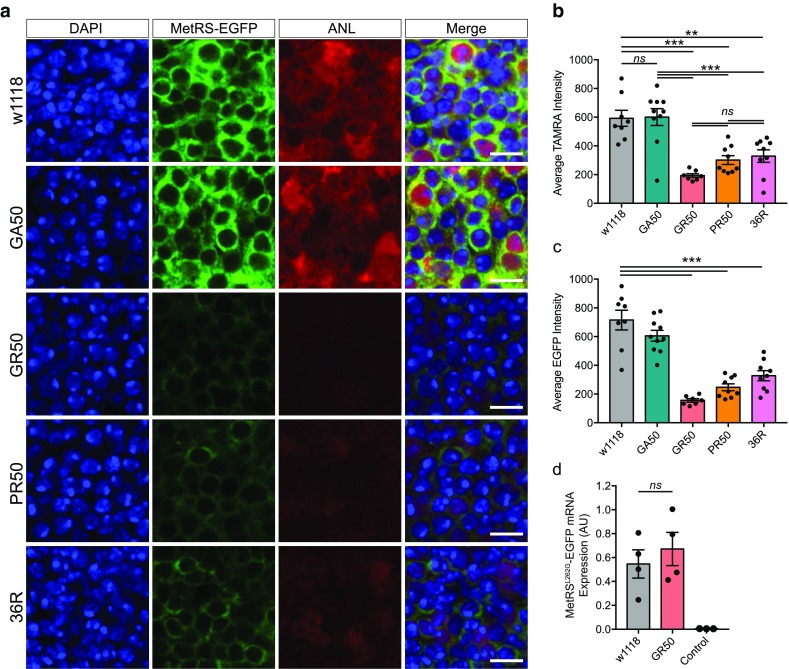
Expression of arginine-rich DPRs suppresses translation in *Drosophila* models. **a** Representative images showing expression of UAS-MetRS^L262G^-EGFP (green) as well as incorporation of ANL (red), DAPI is shown in blue. Scale bar = 5 μm **b** Quantification of ANL incorporation (measured as intensity of TAMRA labelling) for each line. ANL incorporation was reduced compared to controls (w1118) in flies expressing GR50 (one way ANOVA *P* < 0.0001, ***P < 0.0001, Tukey’s multiple comparison test), PR50 (***P = 0.0003) and 36R (**P = 0.0011), but not GA50-expressing flies (*ns* not significant, *P* = 0.9998). Translation was reduced compared to GA50-expressing flies in flies expressing GR50 (***P < 0.0001), PR50 (***P = 0.0002), and 36R (***P = 0.0009). No significant differences were observed between GR50, PR50 and 36R (*ns* not significant). Bars are mean ± SEM, points represent individual brains (*n* = 7–10 per genotype). **c** MetRS^L262G^-EGFP intensity was reduced in flies expressing GR50, PR50 and 36R (one way ANOVA *P* < 0.0001, Dunnett’s test ***P = 0.0001), but not GA50 (*P* = 0.1747). Bars are mean ± SEM, points represent individual brains (*n* = 7–10 per genotype). **d** RT-qPCR data demonstrated that transcript abundance of MetRS^L262G^-EGFP was not significantly lower in flies expressing GR50 compared to controls (w1118) (*ns* not significant, *P* = 0.5188, two-tailed *t* test). Expression of EGFP was not observed in flies not carrying the MetRS^L262G^-EGFP transgene (control). Bars are mean ± SEM, points represent individual samples (*n* = 4 for w1118 and GR50 samples, *n* = 3 for control). Genotypes: w; +; +/elavGS, MetRS^L262G^-EGFP (w1118), w; +; UAS-GA50-FLAG/elavGS, MetRS^L262G^-EGFP (GA50), w; +; UAS-GR50-FLAG/elavGS, MetRS^L262G^-EGFP (GR50), w; +; UAS-PR50-FLAG/elavGS, MetRS^L262G^-EGFP (PR50), w; +/UAS-36R; +/elavGS, MetRS^L262G^-EGFP (36R), w; +; +/TM3,sb (control)

To determine whether translation was repressed in an in vivo model system, we adapted a recently published system for fluorescent non-canonical amino acid tagging (FUNCAT) in *Drosophila* [9]. This method is based on delivery of the non-canonical amino acid Azidonorleucine (ANL), which can only be incorporated into nascent proteins in cells overexpressing the UAS-MetRS^L262G^ methionyl-tRNA synthetase, with subsequent assessment of translation rates by fluorescently labelling the newly synthesised proteins with a tetra-methyl rhodamine (TAMRA) tag using click-chemistry. We recombined the UAS-MetRS^L262G^-EGFP transgene with the elavGS driver, allowing inducible expression specifically in adult neurons. Insertions were backcrossed into the same wild-type stock (w1118) for six generations prior to the experiments, to ensure that observed differences were not due to genetic background. We additionally confirmed that flies expressing arginine-containing DPRs still displayed strong toxicity when expressing the UAS-MetRS^L262G^-EGFP transgene (Suppl. Figure 5, Online Resource 1), whilst flies expressing polyGA had an intermediated lifespan consistent with previous results [6, 28]. To avoid any differences in feeding behaviour affecting results, we directly injected a small volume of ANL into the haemolymph of flies 3 days after induction of expression, before performing dissection and FUNCAT labelling 48 h later. We observed a strong translation repression in flies expressing GR50, PR50 or 36 GGGGCC repeat (36R) constructs compared to controls, but not flies expressing GA50 (Fig. 3a,b). In addition, we also observed a significant reduction in the intensity of the MetRS^L262G^-EGFP enzyme in these flies, (Fig. 3c). We confirmed that this is due to reduced abundance of the MetRS^L262G^-EGFP enzyme using western blotting in whole head lysates (Suppl. Figure 6, Online Resource 1). Despite this reduction, we did not observe a reduction in MetRS^L262G^-EGFP transcript abundance by RT-qPCR (Fig. 3d), demonstrating that translation of the MetRS^L262G^-EGFP enzyme was reduced as part of the translational repression induced by the arginine-rich DPRs, rather than a suppression of transgene expression per se, an effect that has been previously reported [32]. These results show that the arginine-containing DPRs suppress translation in neurons in vivo.

### Eukaryotic initiation factor 1A expression rescues *C9orf72* toxicity in *Drosophila*

**Fig. 4:**
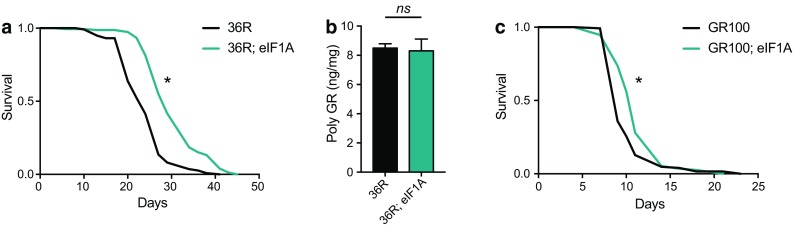
Overexpression of eIF1A rescues toxicity in *Drosophila* models independently of polyGR expression. **a** Lifespan of flies expressing 36 repeats alone (36R) or with the UAS-eIF1A transgene (36R; eIF1A) using the elavGS driver. Lifespan is significantly extended in flies expressing 36R with overexpression of eIF1A compared to 36R alone (median lifespan 36R = 23.0 days, 36R; eIF1A = 28.0, *P = 1.15E − 16, log rank test). **b** Expression of polyGR was not reduced in flies expressing 36 repeats with eIF1A (36R; eIF1A) compared to 36 repeats alone (36R) (*P* = 0.8315, two-tailed *t* test, *ns* not significant). Bars are mean ± SEM (*n *= 4 samples per genotype). **c** Lifespan was significantly extended in flies expressing GR100 with overexpression of eIF1A (GR100; eIF1A) compared to GR100 alone (GR100) (median lifespan GR100 = 8.0 days, GR100; eIF1A = 10.5 days, *P = 9.35E−05, log rank test). Genotypes: w; UAS-36R/+; elavGS/+(36R), w; UAS-36R/+; elavGS/UAS-eIF1A (36R; eIF1A), w; UAS-GR100/+; elavGS/+ (GR100), w; UAS-GR100/+; elavGS/ UAS-eIF1A (GR100; eIF1A)

We determined whether genetic interventions that could enhance translation can rescue toxic phenotypes associated with *C9orf72* repeat RNA expression in vivo. We first attempted to rescue the lifespan phenotype previously reported in a 36 GGGGCC (36R) repeat-expressing *Drosophila* model [28] by overexpressing factors potentially regulating translation. We carried out a mini-screen looking at 70 ribosomal proteins and 11 translation initiation factors (available through the FlyORF library). These were individually expressed in adult *Drosophila* neurons co-expressing 36R using the elavGS driver (Suppl. Figure 7, Online Resource 1). Lifespans were run in batches, always with a wild-type control. The lifespans were scored until the 30–40% of controls had died, so we would be able to identify both factors rescuing and enhancing toxicity. The total number of dead flies in each condition was then counted and expressed as a percentage relative to the control line. Seven candidates that showed a number dead that was < 30% of the control population were considered as rescuers. To rule out a genetic background effect, these stocks were rescreened after being backcrossed, and only a single protein, eukaryotic translation initiation factor 1A (eIF1A), which is essential for translation initiation [8], was able to reduce toxicity in flies expressing 36 repeats (Fig. 4a). This reduction in toxicity was not associated with reduced levels of the toxic GR protein (Fig. 4b), demonstrating that eIF1A overexpression did not affect RAN translation or polyGR clearance. Consistent with this, overexpression of eIF1A also partially rescued the lifespan defect observed in flies expressing GR100 in adult neurons (Fig. 4c). Consistent with a shared mechanism of toxicity, overexpression of eIF1A also extended lifespan in PR100-expressing flies (Suppl. Figure 8a, Online Resource 1). However, neuronal overexpression of eIF1A alone resulted in a modest shortening of lifespan, suggesting that eIF1A does not improve general neuronal health independently of arginine rich-DPR expression (Suppl. Figure 8b, Online Resource 1). These results strongly implicate a conserved regulator of translation as a key modulator of the toxicity observed in an animal model of the *C9orf72* repeat expansion.

### Eukaryotic initiation factor 1A expression rescues translation defects in cells expressing polyGR

**Fig. 5:**
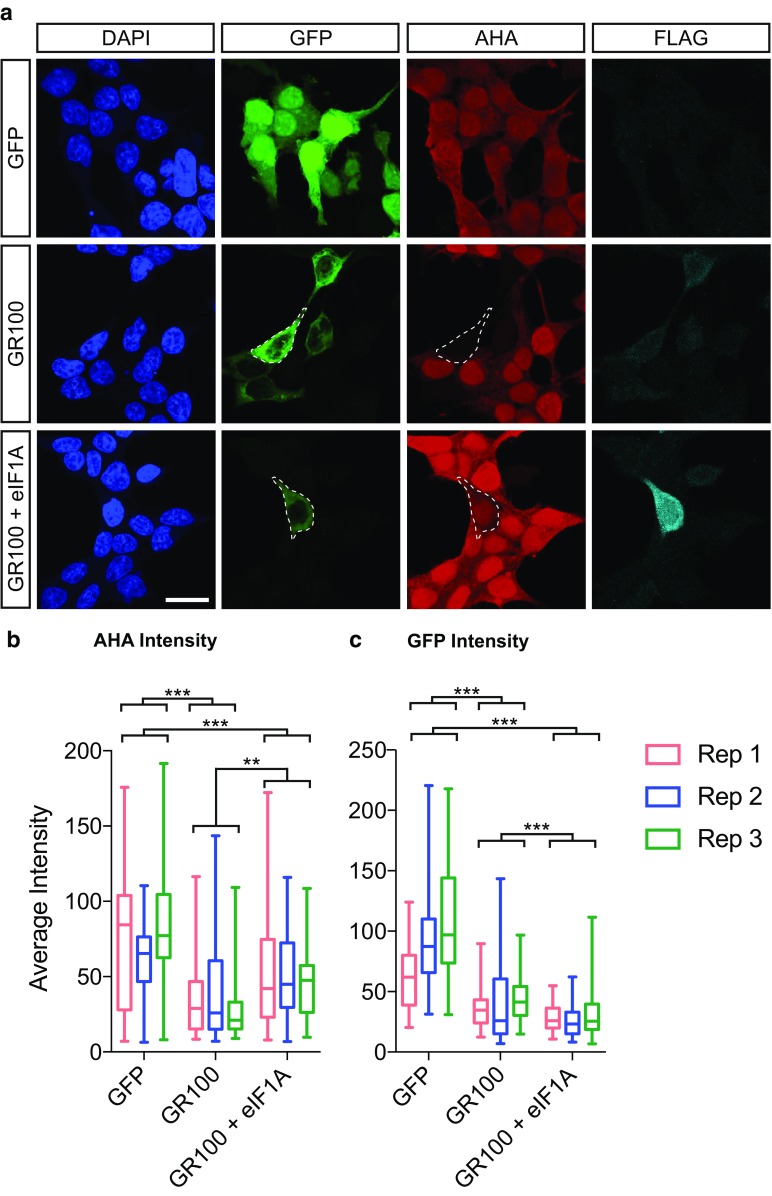
Overexpression of eIF1A reduces translational repression in human cells. **a** HeLa cells were transfected with plasmids encoding GFP alone (GFP), GFP-GR100 with a FLAG tag encoding vector, or GFP-GR100 with FLAG-tagged EIF1AX (GR100 + eIF1A). Protein synthesis was monitored using AHA (red), whilst expression of constructs was measured using GFP expression (green), and immunostaining for FLAG (cyan), cells were counterstained using DAPI (blue). Individual example cells are circled (dotted lines). Scale bar = 20 µm. **b** Quantification of the average intensity of AHA per cell (box plot showing interquartile range with minimum and maximum values). Three independent experimental replicates are shown (Rep 1, Rep 2, Rep 3). A linear model was fitted to the data with technical covariate (day of the experiments) and treatment as fixed effects. We considered three pairwise comparisons between the three treatment groups, controlling for multiple testing using Tukey’s post hoc procedure. Both GR100 and GR100 +eIF1A were highly significant compared to GFP (adjusted ***P < 10^−10^). GR100 + eIF1A also had a higher mean than GR100, with a more modest significance level (adjusted **P = 0.0011). **c** Quantification of the average intensity of GFP per cell (box plot showing interquartile range with minimum and maximum values). Three independent experimental replicates are shown (Rep 1, Rep 2, Rep3). Statistics were performed as in b. Three pairwise comparisons were performed between groups controlling for multiple testing using Tukey’s post hoc procedure. Both GR100 and GR100 +eIF1A were highly significant compared to GFP (adjusted ***P < 10^−10^). GR100 + eIF1A had a higher mean than GR100 (***P = 0.0009). *n* (cells) total, GFP = 305, GR100 = 173, GR100 + eIF1A = 168

We next sought to determine if overexpression of eIF1A enhances translation in polyGR-expressing cells. As the FUNCAT *Drosophila* system was not suitable (see discussion), we assessed translation in a simpler human cell model. We overexpressed GFP-GR100 in HeLa cells and again observed a strong reduction in translation using the AHA assay compared to controls expressing GFP alone (Fig. 5a, b). We cloned the human ortholog of eIF1A (*EIF1AX*) with an N-terminal FLAG tag. When human eIF1A is co-expressed, we observe a significant increase in translation compared to cells co-expressing GFP-GR100 with FLAG alone (Fig. 5b). Although we observed an increased translation in eIF1A-expressing cells, we also observe a slight reduction in the GFP-GR100 signal (Fig. 5b), which may be responsible for this rescue (see discussion). Overall, these data implicate eIF1A, a potential inducer of translation, as a potent suppressor of GR toxicity in an in vivo model of *C9orf72* repeat expansion toxicity and in human cells.

## Discussion

Here, we have identified DPR-interacting proteins specifically from intact brains of a *Drosophila* hexanucleotide repeat expansion model. We have identified translational factors, including ribosomal proteins as strongly enriched in our datasets. Meta-analysis of other recently produced datasets showed that ribosomal proteins are the most commonly identified interactors of the arginine-rich DPR proteins. We predicted that interaction with the arginine-rich DPRs would induce translational repression, and have observed this in both our *Drosophila* models in vivo as well as in HeLa cells and human iPSC-derived MNs. Further to this, we have demonstrated that overexpression of a key translational regulator can rescue the toxic phenotype observed in our *Drosophila* models. Together, these results strongly support the role of ribosomal dysfunction and translational repression in the pathogenesis of *C9orf72*-associated ALS/FTD.

Alterations in the control of protein translation have been strongly linked to neurodegeneration in number of contexts [17]. TDP-43 and FUS have both been linked to the formation of stress granules and disruption in localised synaptic translation [7]. In addition, translational defects have been linked to a number of ALS-associated diseases, including spinal muscular atrophy (SMA), where SMN protein has been demonstrated to interact with ribosomes, and its loss of function is implicated in inducing translational arrest, particularly in MNs [3, 35]. Additionally, impaired translation has been strongly implicated in the pathogenesis of Charcot–Marie–Tooth (CMT) peripheral neuropathies caused by dominant mutations in several aminoacyl-tRNA synthetases [32]. Finally, tau protein has been shown to bind to ribosomes in Alzheimer’s patient post-mortem tissue and suppress translation in vitro, implicating translational repression in tau-mutation-associated FTD [27].

Previous studies have demonstrated that expression of sense *C9orf72* repeat RNA or exposure of cells to arginine-rich DPRs is sufficient to induce a translational arrest, lending further support to our conclusion that translational arrest represents a key pathological mechanism in the disease state [16, 20, 34]. In other neurodegenerative disorders, such as Alzheimer’s and prion diseases, a potential mechanism of translational arrest is via the phosphorylation of Eif-2-alpha. However, others have shown that DPRs or repeat RNA expression was not sufficient to induce increased Eif-2-alpha phosphorylation [16, 34]. Thus, the mechanism by which translational repression is induced by the arginine-rich DPRs is undetermined, and has previously been suggested to be due to sequestration of translation initiation factors by repeat RNA [34], the direct binding of cellular mRNA by the arginine-containing DPRs [16], or the formation of cytoplasmic stress granules [20]. Whilst other mechanisms are conceivable, multiple studies have demonstrated interaction between the arginine-rich DPRs and ribosomal proteins, even in the presence of RNAse (Suppl. Figure 4, Online Resource 1) [5, 16, 20, 22, 23, 45]. In this regard, it is notable that antimicrobial peptides enriched for PR-containing motifs have been demonstrated to bind to the ribosomal exit tunnel and inhibit bacterial protein synthesis [11]. In support of the ribosome-binding hypothesis, two studies, published after the submission of this article, demonstrate that overexpressed arginine-rich DPRs can inhibit protein synthesis [14, 48]. Strikingly, both studies also identified ribosomal proteins as constituents of polyGR inclusions found in patient brain tissue.

To try and rescue toxicity deriving from this translational defect, we screened 81 *Drosophila* overexpression lines from the FlyORF library. Flies have 79 different cytoplasmic ribosomal proteins [25]. Although we overexpressed the majority of these proteins, we failed to observe a rescue. This is likely due to the fact that ribosomal subunits are large, multi-protein complexes with complex stoichiometry, and thus overexpressing one ribosomal protein would be unlikely to be able to lead to an increase in functional ribosomal subunits and, therefore, to an increase in translation. Flies have 43 translation initiation factors, which have better characterised functions in the regulation of translation [24]. Of the ten that were available to us, one, eIF1A, a conserved component of the pre-initiation complex (PIC), led to a substantial rescue in phenotype when overexpressed. The only other component of the PIC we screened was part of eIF3, which is a large multi-protein complex composed of at least ten non-identical subunits, suggesting that upregulation of one component might not be enough to enhance its activity. It would be interesting, in future, to check whether upregulation of other PIC components can also rescue the translation defect.

We predict that eIF1A directly alleviates the polyGR-induced translation defect. However, with our current methodologies for measuring translation in vivo, we did not detect an increase in global translation in GR100 flies co-expressing eIF1A (data not shown). This is potentially because reduced translation of the MetRS^L262G^-EGFP in polyGR-expressing flies makes it difficult to observe an enhanced translation. Alternatively, it may be because rescue of lifespan can be mediated by enhancing translation in only a subset of neurons, which would not be detected by our global staining approach. Consistent with possibility, DPR-driven toxicity has recently been shown to be selectively toxic to a sub-population of neurons in flies [44]. In support of our proposed mechanism, we observed that overexpression of eIF1A is sufficient to increase translation in human cells. However, in these cells there was a concomitant drop in GFP-GR100 expression, a phenomenon we did not observe in *Drosophila*. Thus, although it is likely that eIF1A overexpression increases translation in flies and human cells, we cannot rule out the possibility that eIF1A rescues *C9orf72* repeat toxicity though a non-canonical mechanism not related to its primary function in translation.

In summary, we observe ribosomal proteins as key interactors of the arginine-rich dipeptide proteins in an in vivo model of *C9orf72*-associated ALS and FTD. We have linked this to a translational repression both observable when arginine-rich DPRs are expressed in HeLa cells, human iPSC-derived MNs and in adult *Drosophila* neurons. Finally, we have identified overexpression of the translation initiation factor eIF1A as a key modifier of toxicity in our *Drosophila* models, further linking the observed translational repression to toxicity.

## Electronic supplementary material

Below is the link to the electronic supplementary material.

Supplementary material 1 (PDF 10030 kb)

Supplementary material 2 (XLSX 101 kb)
