# Supplementary material for: C9orf72 arginine-rich dipeptide proteins interact with ribosomal proteins in vivo to induce a toxic translational arrest that is rescued by eIF1A
Source: Acta Neuropathol. 2019 Jan 2;137(3):487–500. doi: 10.1007/s00401-018-1946-4 (PMC6514073; doi:10.1007/s00401-018-1946-4)
Supplement: Supplementary file 1 — Supplementary material 1 (PDF 10030 kb) [file 401_2018_1946_MOESM1_ESM.pdf]

Online Resource 1

C9orf72 arginine-rich dipeptide proteins interact with ribosomal proteins *in vivo* to induce a toxic translational arrest that is rescued by eIF1A

Thomas G. Moens[1,2,3,4,5], Teresa Niccoli[1,2,3], Katherine M. Wilson[1,3], Magda L. Atilano[1,2,3], Nicol Birsa[6], Lauren M. Gittings[1,3], Benedikt V. Holbling[1,3], Miranda C. Dyson[1,2,3], Annora Thoeng[1,2,3], Jacob Neeves[1,2], Idoia Glaria[1,3], Lu Yu[7,8], Julia Bussmann[9,10], Erik Storkebaum[9,10,11], Mercedes Pardo[7,8], Jyoti S. Choudhary[7,8], Pietro Fratta[12] , Linda Partridge[2,13]*, Adrian M. Isaacs[1,3]*

[1]Department of Neurodegenerative Disease, UCL Institute of Neurology, Queen Square, London WC1N 3BG, UK
[2]Department of Genetics, Evolution and Environment, Institute of Healthy Ageing, UCL, Darwin Building, Gower Street, London WC1E 6BT, UK
[3]UK Dementia Research Institute at UCL, Cruciform Building, London WC1E 6BT, UK
[4]Present address: Present address: KU Leuven – University of Leuven, Department of Neurosciences, Experimental Neurology, and Leuven Brain Institute (LBI), Leuven, Belgium
[5]Present address: VIB, Center for Brain & Disease Research, Laboratory of Neurobiology, Leuven, Belgium
[6]Sobell Department of Motor Neuroscience and Movement Disorders UCL Institute of Neurology, Queen Square, London WC1N 3BG, UK
[7]Wellcome Trust Sanger Institute, Wellcome Trust Genome Campus, Hinxton, Cambridge, United Kingdom
[8]Present address: The Institute of Cancer Research, Division of Cancer Biology, 123 Old Brompton Road, London SW7 3RP
[9]Molecular Neurogenetics Laboratory, Max Planck Institute for Molecular Biomedicine, 48149 Münster, Germany
[10]Faculty of Medicine, University of Münster, 48149 Münster, Germany.
[11]Department of Molecular Neurobiology, Donders Institute for Brain, Cognition and Behaviour, Radboud University, Nijmegen, The Netherlands
[12]MRC Centre for Neuromuscular Disease, UCL Institute of Neurology, Queen Square, London WC1N 3BG, UK
[13]Max Planck Institute for Biology of Ageing, Joseph-Stelzmann-Strasse 9b, 50931 Cologne, Germany

*Correspondence to AMI (a.isaacs@ucl.ac.uk) or LP (linda.partridge@ucl.ac.uk)

Supplementary Methods

Western blotting

Female flies derived from the same cross and induced at the same time as the RT-qPCR flies were induced on SYA medium containing 200 μM RU486 for 5 days before being flash frozen in liquid nitrogen. 7-8 heads per replicate were homogenised in 2x SDS Laemmli sample buffer (4% SDS, 20% glycerol, 120 mM Tris-HCl pH6.8, 200 mM DTT with bromophenol blue) and boiled at 95ºC for 5 minutes. Samples were separated on pre-cast 4-12% Invitrogen Bis-Tris gels (NP0322) and blotted onto PVDF membrane in Tris-glycine buffer supplemented with 10% Ethanol. Membranes were blocked in 5% milk and in TBS-T (TBS with 0.05% Tween-20) for 1 hour at room temperature and then incubated with primary antibodies in TBS-T. Primary antibody dilutions used were: anti-GFP 1:1000 (Invitrogen A-11122), anti-actin 1:10,000 (Abcam ab1801). Secondary antibodies were: HRP conjugated anti-rabbit and anti-mouse (Abcam ab6789 and ab6721) at 1:10,000 dilution for 1 hour at RT. Bands were visualized with Luminata Forte (Millipore) and imaged with ImageQuant LAS4000 (GE Healthcare Life Sciences). Quantification was carried out with ImageQuant software.

Dot Blot

Female flies were induced on SYA medium containing 200 μM RU486 for 5 days before being flash frozen in liquid nitrogen. 7-8 heads per replicate were homogenised in 2x SDS Laemmli sample buffer (4% SDS, 20% glycerol, 120 mM Tris-HCl pH6.8, 200 mM DTT with bromophenol blue) and boiled at 95ºC for 10 minutes and centrifuged for 10 minutes at 15,000 rpm. 10 μl of extract was blotted onto nitrocellulose, allowed to dry for 10 minutes, blocked in 5% milk in TBS-T (TBS with 0.02% Tween-20) for 1 hour at room temperature. To visualise the protein G tag, the blot was incubated with HRP conjugated anti-rabbit antibody (Abcam ab6789) in TBS-T for 1 hour. Blots were imaged as described for Western Blot.

Suppl. Figure 1

a

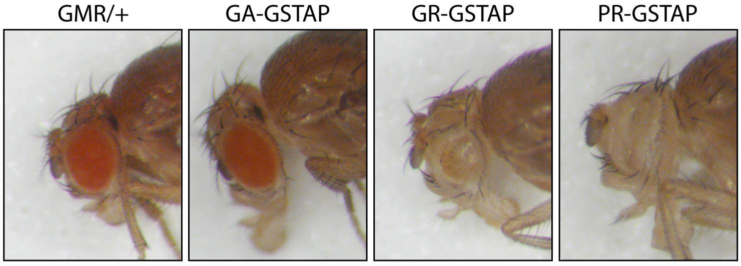

b

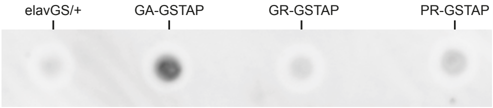

Suppl. Figure 1 a Arginine-containing dipeptide proteins are toxic in the presence of the GSTAP tag. Flies were crossed to the GMR-gal4 driver and no overt rough eye was visible in flies carrying the driver alone (GMR/+) or flies expressing the GA-GSTAP control construct. Severe toxicity was observed in GR-GSTAP and PR-GSTAP expressing flies. Genotypes: w; GMR-gal4/+ (GMR/+), w; GMR-gal4/UAS-GA-GSTAP (GA-GSTAP), w; GMR-gal4/UAS-GR-GSTAP (GR-GSTAP), w; GMR-gal4/+; UAS-PR-GSTAP/+ (PR-GSTAP). **b** Expression of GSTAP-tagged dipeptide proteins was assessed by dot blot, expression of GR-GSTAP and PR-GSTAP was lower that GA-GSTAP. Genotypes: w; +; elavGS/+ (elavGS/+), w; UAS-GA-GSTAP/+; elavGS/+ (GA-GSTAP), w; UAS-GR-GSTAP/+; elavGS/+ (GR-GSTAP), w; + ; UAS-PR-GSTAP/elavGS (PR-GSTAP).

Suppl. Figure 2

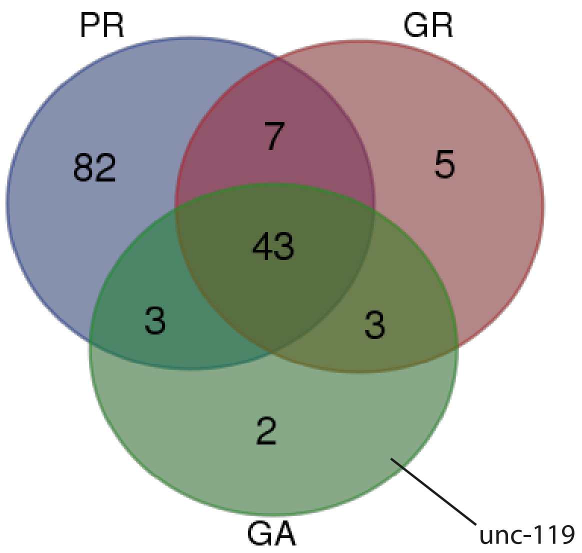

Suppl. Figure 2 Complete data set from all dipeptide proteins. Numbers represent individual proteins identified in a minimum of 2/3 replicates. A consistently larger number of interactors were identified as binding specifically to PR (82/94), compared to GR, where (5/94) interactors were specific to GR, and (7/94) interactors were specific to both data sets. Any proteins that bound to GA in ≥2/3 replicates were not considered in the analysis of PR and GR interacting proteins (49 proteins total). Of note, only two proteins were identified as binding to GA, one of which was unc-119 which has previously been identified as a GA interacting protein [26].

Suppl. Figure 3

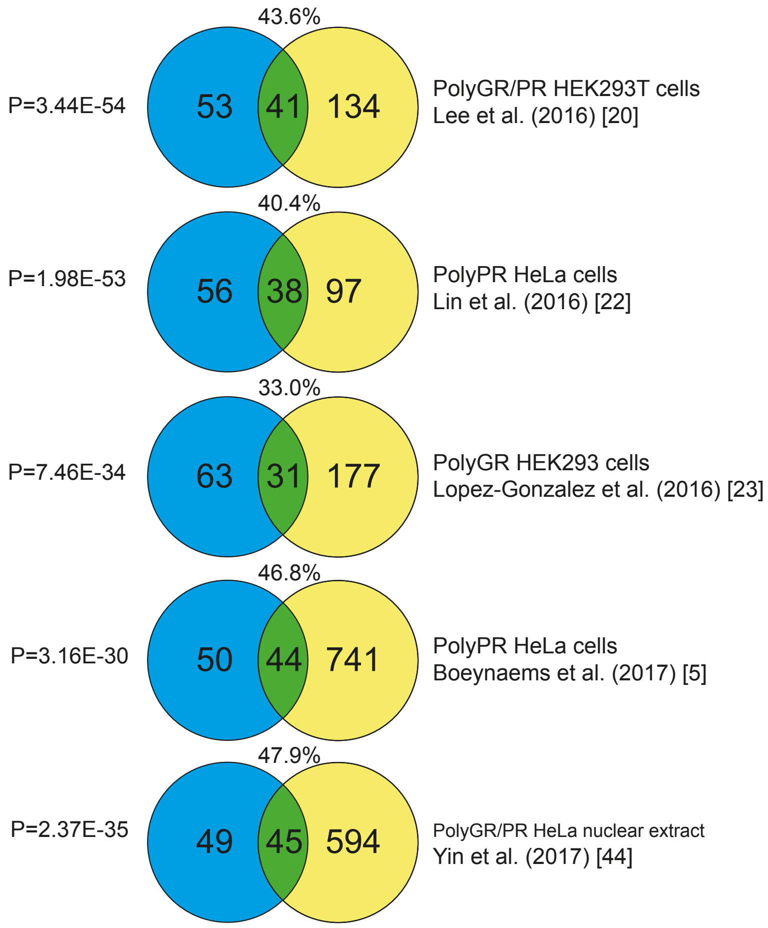

Suppl. Figure 3 Overlap of *Drosophila* arginine-DPR interactome data with previously published data sets. The indicated polyGR and/or polyPR human data sets were converted to *Drosophila* orthologs (yellow) and compared to the 94 identified *Drosophila* proteins (blue). Cell type and purified dipeptide protein are given. Overlaps are given both numerically, or as a % overlap with the *Drosophila* data set. A highly significant overlap was observed with all published data sets (P value is the probability of observing the number of overlapping interactions or more by chance, assessed using the hypergeometric test, based on a *Drosophila* genome size of 13,931 protein coding genes).

Suppl. Figure 4

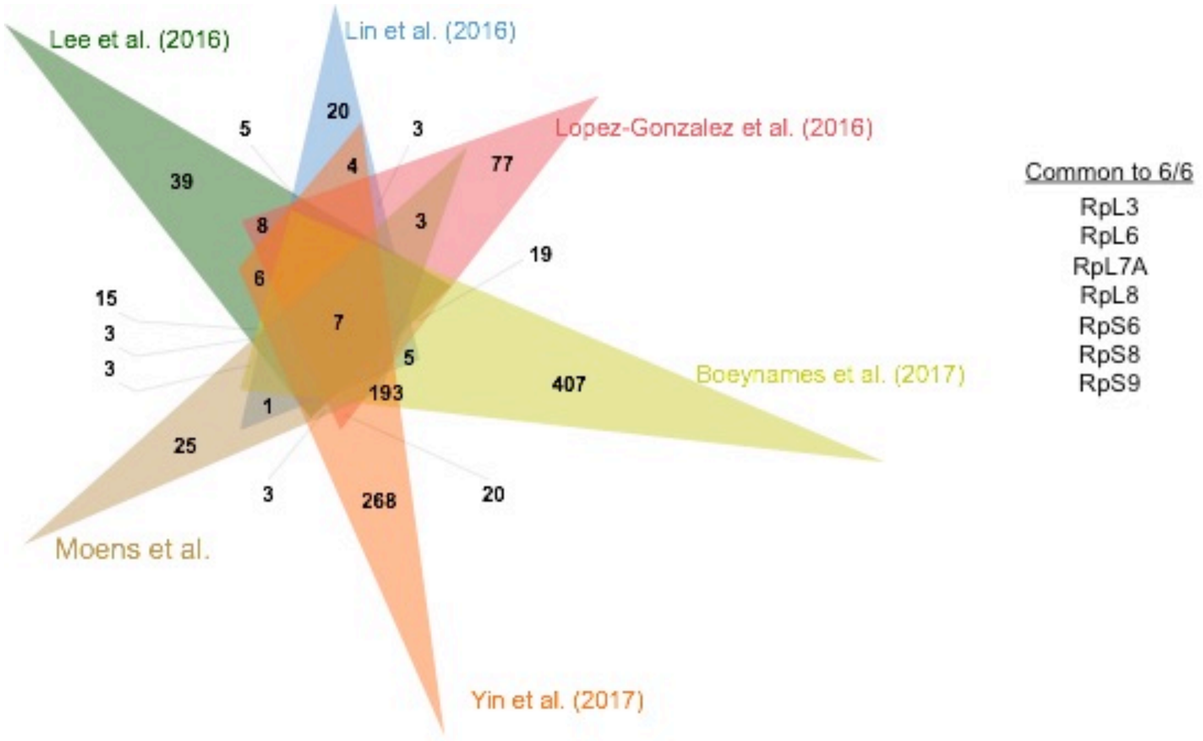

Suppl. Figure 4 Venn diagram representing the overlap of six published mass spectrometric data sets compared to the Drosophila dataset (Moens et al.). 7 proteins were common as orthologs between all data sets (6/6). Notably, these were all ribosomal proteins from both the large and small ribosomal subunits.

Suppl. Figure 5

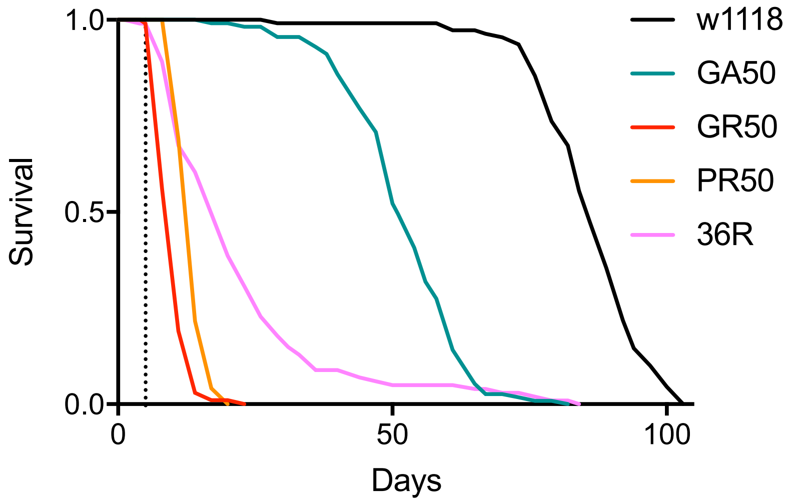

Suppl. Figure 5 Lifespans of flies expressing DPR or repeat constructs when coexpressed with MetRS^{L262G}-EGFP. A significant reduction in lifespan was observed in flies expressing GR50-FLAG and PR50-FLAG compared to controls carrying the driver and MetRS^{L262G}-EGFP alone (w1118) (P=3.89E-55 and 4.89E-54 respectively, log rank test). A significant reduction in lifespan was observed in flies expressing $(GGGGCC)_{36}$ (36R) vs. w1118 (P=7.51E-55, log rank test). A smaller reduction in lifespan was observed in flies expressing GA50 vs. w1118 (P=1.61E-53). The dashed line at day 5 represents the time point that flies were dissected in FUNCAT experiments. Median lifespans: w1118=85.0 days, GA50=50.5 days, GR50=9.5 days, PR50=12.5 days, 36R=15.5 days. Genotypes: w; +; +/ elavGS, MetRS^{L262G}-EGFP (w1118), w; +; UAS-GA50-FLAG/ elavGS, MetRS^{L262G}-EGFP (GA50), w; +; UAS-GR50-FLAG/ elavGS, MetRS^{L262G}-EGFP (GR50), w; +; UAS-PR50-FLAG/ elavGS, MetRS^{L262G}-EGFP (PR50), w; +/UAS-36R; +/elavGS, MetRS^{L262G}-EGFP (36R).

Suppl. Figure 6

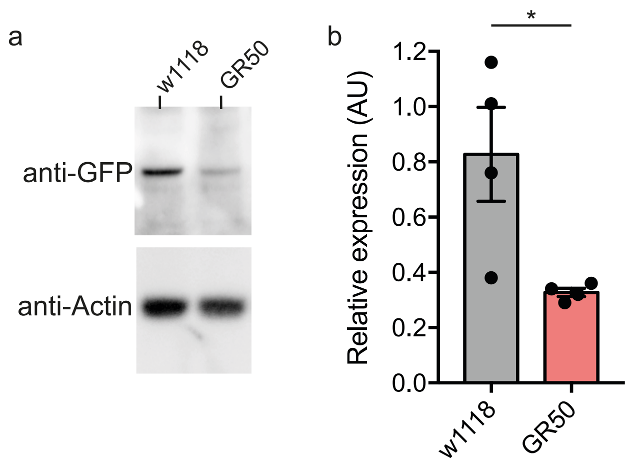

Suppl. Figure 6 Immunoblotting confirms reduced abundance of the MetRS^{L262G}-EGFP enzyme in flies expressing GR50 compared to controls. **a** Blot demonstrating reduced abundance of the MetRS^{L262G}-EGFP enzyme (detected using an anti-GFP antibody) in flies expressing GR50 compared to the enzyme alone. Actin loading control is shown. **b** Quantification of MetRS^{L262G}-EGFP enzyme levels using western blotting. A significant reduction in MetRS^{L262G}-EGFP protein abundance was detected in GR50 expressing flies compared to flies expressing the transgene alone (w1118) (*P=0.0286, two-tailed Mann Whitney test). Bars are mean ±SEM, individual data points are shown. n=4 samples per genotype. Genotypes: w; +; +/ elavGS, MetRS^{L262G}-EGFP (w1118), w; +; UAS-GR50-FLAG/ elavGS, MetRS^{L262G}-EGFP (GR50).

Suppl. Figure 7

Translation Factor	Lifespan effect	Re-screen
RpL18A		X
eIF1A		YES
eIF4H1		X
RpS20		X
RpL35A		X
RpS5b		X
RpS15Aa		X
RpL40		
RpL27A		
RpS21		
RpL6		
RpS15		
RpLP2		
RpL36A		
RpS8		
RpL39		
RpL32		
RpL12		
RpL34b		
RpS28b		
RpL41		
RpL31		
RpL27		
RpL26		
RpS14b		
RpS16		
RpS11		
RpS10a		
RpL11		
RpLP0		
RpL24		
RpS27A		
RpS18		
RpL14		
RpS26		
RpS13		
eIF4AIII		
RpL21		
RpS19b		
RpS27		
RpL18		

Translation Factor	Lifespan effect	Re-screen
RpL10		
RpL34a		
RpS3		
RpS15Ab		
RpL4		
eIF2α		
RpL19		
Tango7		
RpL8		
RpL13A		
RpS10b		
RpL13		
RpL30		
RpL17		
RpS17		
RpL35A		
RpL38		
RpS7		
RpLP1		
eIF4E6		
RpL9		
eIF3d1		
RpS25		
RpL23A		
eIF4A		
RpL22		
RpL10Ab		
RpL3		
RpL37b		
RpS30		
RpL7		
RpL7A		
eIF4E1		
RpS23		
RpL15		
RpL5		
RpL7-like		
RpL36		
CG9769		
CG8636		

Key
<30%
30-60%
60-90%
90-120%
120-150%
150-180%
180-210%
210-240%
>240%

Suppl. Figure 7 List of translation-associated proteins checked for the effect of their overexpression on the lifespan of the UAS-36R expressing flies. Colours indicate the degree of change in lifespan relative to the control background strain (see key). Seven strong suppressors were found to have a lifespan reduction <30% of control (dark green), and were backcrossed into a control (w1118) genetic background for 6 generations prior to being tested again (indicated in the re-screen column). Of these 7 lines, only eIF1A extended lifespan after accounting for genetic background (see Figure 4). Genotypes: w; UAS-36R/+; UAS-indicated-line /elavGS (indicated lines), w; UAS-36R/+; attP-86Fb/ elavGS (control).

Suppl. Figure 8

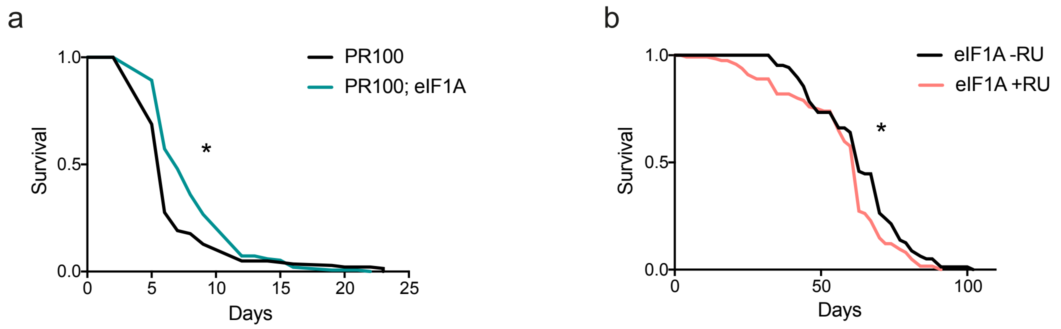

Suppl. Figure 8 Overexpression of eIF1A is specifically protective in flies expressing arginine DPRs. **a** Lifespan of flies expressing PR100 alone (PR100) or with the UAS-eIF1A transgene (PR100; eIF1A). Lifespan is significantly extended in flies expressing PR100 with overexpression of eIF1A compared to PR100 alone (median lifespan PR100=5.5 days, PR100; eIF1A=6.5, *P=2.08E-4, log rank test). **b** Lifespan of flies carrying the UAS-eIF1A construct and elavGS driver. Flies were either fed RU486 to induce expression (eIF1A +RU), or control food (eIF1A –RU). Lifespan was significantly shorter in flies expressing eIF1A (eIF1A +RU), compared to controls (eIF1A –RU) (median lifespan +RU= 61.5 days, -RU= 61.5 days, *P=0.017, log rank test). Genotypes: w; UAS-PR100/+; elavGS/+ (PR100), w; UAS-PR100/+; elavGS/UAS-eIF1A (PR100; eIF1A), w; + ; elavGS/UAS-eIF1A (eIF1A -RU/+RU).
